# The burden of insomnia among public health sector nurses in KwaZulu-Natal, South Africa

**DOI:** 10.4102/sajpsychiatry.v31i0.2433

**Published:** 2025-05-22

**Authors:** Phatheka Patience Ntaba, Sibongile Mashaphu, Kalpesh Narsi

**Affiliations:** 1 Discipline of Psychiatry, School of Clinical Medicine, University of Kwazulu-Natal, Durban, South Africa

**Keywords:** insomnia, nurses, health care workers, sleep hygiene, burnout

## Abstract

**Background:**

Insomnia is defined as poor quantity or quality of sleep resulting in impaired daytime functioning and distress. Insomnia has been found to occur at higher rates in health care workers and has been associated with physiological dysfunction, illness and distress as well as many socio-demographic and lifestyle-related factors.

**Aim:**

This study aims to establish the burden of insomnia and its associated socio-demographic, lifestyle and occupation-related factors among nurses.

**Setting:**

Nurses employed in the public sector in Kwazulu-Natal Province South Africa comprised the study group. A cross-sectional descriptive online survey was conducted.

**Methods:**

Nurses were invited to participate in an online survey from September to December 2023. The survey comprised a socio-demographic questionnaire and three instruments: the Sleep Condition Indicator, the Single Item Measure of Burnout and the Sleep-Hygiene Index (SHI).

**Results:**

Of the 235 participants surveyed, 41.7% screened positive for insomnia and had significantly lower frequencies of good sleep-hygiene practices on the SHI. After controlling for socio-demographic, clinical and work-related covariates, a psychiatric history adjusted odds ratio [aOR]: 5.52; CI:1.06–28.66) and poor sleep hygiene (aOR: 1.07; CI: 1.02–1.13) displayed significant association with insomnia. All levels of work-related stress were significantly associated with insomnia (*p* < 0.01), with total burnout having a 10.3-fold increased association.

**Conclusion:**

The study highlights the concerning burden of insomnia and its association with burnout, poor sleep hygiene and a psychiatric comorbidity, among nurses.

**Contribution:**

Given that the risk factors associated with insomnia identified in our study (i.e. poor sleep hygiene, burnout and psychiatric illness) are all potentially modifiable, our findings may serve as a reference for future health-promotion initiatives, aimed at health care professionals, such as health education, screening and mindfulness and wellness programmes.

## Introduction

Sleep plays a crucial role in both physical and mental homeostasis,^1^ with the quality of sleep having a significant impact on cognitive functioning and emotional stability.^2^ Good sleep enables individuals to be alert and engage productively in their social and occupational roles, whereas inadequate sleep hinders this capacity and has been associated with numerous adverse physical and mental consequences, impairment in quality of life (QoL) and higher levels of perceived stress.^3^

Insomnia may be defined as a collection of symptoms where individuals experience challenges in initiating, maintaining and/or returning to sleep, causing significant day-time dysfunction.^4^ The prevalence of the disorder of insomnia, as classified in the Diagnostic and Statistical Manual 5th edition, text revised (DSM-5TR), is around 4% – 22% with an average of 10%.^5^ Globally, 10% – 40% of the general population suffers from insomnia,^6^ while in South Africa, this figure is reported to be between 8% and 44%.^7^

Health care workers appear to be at particular risk of insomnia, having a much higher rate of 30% – 90% of this condition globally.^3^ During the coronavirus disease 2019 (COVID-19) epidemic, an in-depth review revealed that nurses exhibited the highest rate of insomnia compared to all other health care workers.^8^ Using the Insomnia Severity Index, a tool with less stringent criteria than the DSM-5, a Kenyan study found the prevalence of insomnia in nurses to be 41.1%.^9^

Serving a population approaching 70 million,^10^ the South African health system continues to be a strained sector with a shortage of nurses and an overcrowded health care system, which has been shown to be associated with longer working hours and burnout.^11^ Insomnia may impair nurses’ attention and memory, causing emotional instability, decreased productivity, absenteeism and other psychological issues.^12^ Insomnia leading to fatigue may cause medical errors at work and job dissatisfaction, thus affecting patient care and leading to medico-legal consequences.^13^ This therefore underscores the need for health care worker wellness, which includes appropriate sleep hygiene and low levels of insomnia.

A variety of socio-demographic and lifestyle-related factors may influence sleep patterns, with age and gender being the most common risk factors, and females and the elderly displaying a higher prevalence.^14^ Among nurses, numerous studies have associated insomnia with work stress, burnout, gender, employment experience, chronic illnesses, midday nap duration and shift work.^12,15,16^ While comorbid disorders, psychiatric conditions and working night shifts have been significantly associated with insomnia, these factors may not be causal but may rather serve as precipitants to insomnia.^17^

Poor sleep hygiene practices are also significantly associated with poor sleep quality and may harm mental and physical health and lower QoL in health care shift workers.^18^ Health care workers have been found to have poor sleep hygiene, for example daytime napping and increased caffeine use, compared to the general population.^19^ Considering the fact that nurses often play a crucial role in health promotion and patient counselling, it is important to investigate their own health-promoting practices such as sleep hygiene.

Given the general finding that nurses appear to be at particular risk of insomnia, and the extent to which the South African public health system depends on nursing staff in particular, this study aims to determine the burden of insomnia and its association with socio-demographic profile, occupational and lifestyle-related factors among nurses in KwaZulu-Natal (KZN) Province, South Africa. At the time of writing, there is a paucity of studies on insomnia in this population in South Africa.

## Research methods and design

This is a cross-sectional, descriptive quantitative study comprising nurses currently working in public health facilities (clinics and hospitals) in KZN, South Africa. Nurses who are registered with the South African Nursing Council (SANC), between the ages of 18 and 60 years and employed in the public sector were invited to participate in the study. Non-public sector nurses, non-nursing medical professionals and those with sensory-motor deficits or acute medical illnesses were excluded. A minimum sample size of 215 was required, calculated using an online sample size calculator (www.calculator.net/sample-size-calculator.html) with a 95% confidence level, a 41% estimated population proportion based on a previous study^9^ and a 10% margin of error. To account for a potential non-response rate of 50%, the final sample size was adjusted to 235.^20^ Of the 260 responses received, 25 participants were excluded owing to withdrawal from the survey or not meeting inclusion criteria (e.g. nurses employed in the private sector and other health professionals). A final sample size of 235 nurses was attained. Data collection was conducted using convenience sampling on REDCap (Research Electronic Data Capture), a secure web tool for creating and maintaining online surveys and databases for research and operations. Between September and December 2023, nurses were invited to participate in the survey via a weblink, which was shared through email, a notice on the Department of Health’s website and social media platforms.

### Measures

Participants were administered four instruments: a researcher-designed socio-demographic questionnaire, the Sleep Condition Indicator (SCI), Single Item Measure of Burnout (SIMB) and Sleep Hygiene Index (SHI). The socio-demographic questionnaire included demographic, biographical, clinical and lifestyle-related questions, developed by the researchers and piloted on a sample of 10 nurses to assess its feasibility prior to implementation. Factors examined were age, race, gender, nursing category, workplace, years of experience, workplace support, absence because of insomnia, substance use, family history and medical history. The SCI is based on DSM-5 criteria for primary insomnia^21^ and is an eight-item scale with the potential range of score from 0 to 32, with higher values indicating superior sleep quality.^21^ A cutoff score of 16 was used, with individuals scoring below 16 being regarded as positive for insomnia disorder. The scale exhibited strong internal consistency as indicated by a Cronbach’s alpha coefficient of 0.86 or higher and demonstrated convergent validity. While the SCI had not been validated in South Africa, no other instrument was identified, which utilises the DSM-5 diagnostic criteria for insomnia disorder.

The SIMB, developed by Schmoldt et al., is a single-item rating of subjective burnout, with responses range from 1 (*I feel extremely exhausted and question my ability to continue. I may need to make changes or seek help*) to 5 (*I enjoy my work. I have no burnout symptoms*).^22^ Research into its validity showed this scale to be conceptually similar and substantially congruent with the Maslach Burnout Inventory (MBI).^23^ Compared to the MBI:EE (emotional exhaustion), the non-proprietary single-item measure exhibited a correlation of 0.79, sensitivity of 83.2%, specificity of 87.4% and area under the curve of 0.93 (s.e. = 0.004).^24^ This questionnaire has also not been validated in South Africa.

The SHI is a 13-item self-reported measure developed from the International Classification of Sleep Disorders (ICSD) to evaluate sleep hygiene behaviours.^25^ For each item, the frequency of sleep-promoting behaviour engaged in by the respondent is rated on a five-point Likert scale ranging from 0 ‘never’ to 4 ‘always’.^25^ The total score ranges from 0 to 52, with higher scores suggesting less-than-optimal sleep hygiene.^25^ The SHI has a higher Cronbach’s α (α = 0.66) than earlier instruments and demonstrated strong test–retest reliability (*r* (139) = 0.71, *p* < 0.01),^26^ the tool also not having been validated in South Africa.

### Data analysis

IBM SPSS Statistics version 28 for Windows was utilised for statistical analysis. Continuous variables were found to be normally distributed using the Shapiro–Wilk test and visual inspection of histogram plots and were summarised using means and standard deviations (s.d.). Categorical variables were described using proportions (%), and Pearson’s chi-square test was performed to measure the association between categorical variables. Assumptions for the chi-square test were checked using Fisher’s exact test, and with *p*-values, odds ratios and 95% confidence intervals were reported. To account for potential confounders, variables with a *p* < 0.3 in the univariate analysis were included in the multivariate logistic regression model. This threshold was chosen to ensure that variables with possible confounding effects were not prematurely excluded.^27^ A *p*-value of less than 0.05 was considered statistically significant.

### Ethical considerations

Ethical clearance to conduct this study was obtained from the University of Kwazulu-Natal Biomedical Research Ethics Committee (No. BREC/00005346/2023) and the KwaZulu-Natal Department of Health. All participants were provided with study information on an online platform before providing informed consent. Confidentiality was maintained through the authors having exclusive access to the data and no identifying data being used.

## Results

Four sets of results are presented: those relating to the socio-demographic questionnaire and SIMB, the SCI and clinical factors, the SHI, and a multi-variate regression to establish association.

### Participants’ characteristics

A total of 235 completed surveys were received. The participants’ socio-demographic and occupational characteristics are displayed in Table 1.

**TABLE 1:** Socio-demographic and occupational profile of participants (*N* = 235).

Variable	*n*	%
**Gender**		
Female	182	77.40
Male	53	22.60
**Age (years)**		
22–30	21	8.90
31–40	78	33.20
41–50	90	38.30
51–60	46	19.60
**Race**		
Black people	196	84.50
Coloured people	7	3.00
Indian people	27	11.60
White people	2	0.90
Not reported	3	1.30
**Marital status**		
Single and/or divorced	100	42.60
Married and/or cohabiting	120	51.10
Widowed	15	6.40
**Education level**		
Nursing certificate	46	19.60
Diploma and/or bridging	135	57.40
Bachelors	26	11.10
Masters and/or speciality	28	11.90
**Occupational category**		
Enrolled nurse assistant	49	20.90
Staff nurse	25	10.60
Professional nurse	141	60.00
Manager	20	8.50
**Work environment**		
Office-based and/or administrative	14	6.10
Outpatient	41	18.00
Inpatient	173	75.90
Not reported	7	3.00
**Employment duration**		
≤ 24 months	32	14.00
> 24 months	197	86.00
Not reported	6	2.60
**Night shift work**		
No	63	27.50
Yes	166	72.50
Not reported	6	2.60
**Work absenteeism because of insomnia**		
No	210	89.40
Yes	25	10.60
**Work-related burnout (SIMB category)†**		
1	18	7.70
2	37	15.70
3	58	24.70
4	61	26.00
5	61	26.00

*Source:* Schmoldt R, Freeborn D, Klevit H. Physician burnout: Recommendations for HMO managers. HMO Pract. 1994;8(2):58–63.

Note: Likert range: 1 = Total burnout; 5 = No burnout.

† , Single item measure of burnout (Likert range: 1 = total burnout; 5 = no burnout).

Participants’ clinical profiles, including substance and medication use, are presented in Table 2, with 41.7% screening positive for insomnia using an SCI cut-off score of 16. A positive family history of insomnia was reported by 66 individuals (28.2%), while co-morbidities were reported by 17 (7.2%) having been diagnosed with a psychiatric disorder and 70 (29.8%) having a general medical condition (e.g. hypertension, diabetes, epilepsy and allergies). In addition, 28 (12%) participants reported using alcohol, two (0.9%) used cannabis to assist with sleep and 20 (8.6%) used nicotine in the form of cigarettes; 49 (20.9%) did not consume caffeine-containing beverages such as coffee or energy drinks; 140 (59.8%) consumed 1–2 cups of caffeinated-drinks daily and 45 (19.2%) consumed more than two cups daily. Regarding medication used for insomnia, 48 (20.4%) utilised prescription sedatives (the commonest being amitriptyline, followed by alprazolam and zolpidem), and an additional 85 (36.2%) used over-the-counter drugs (including non-prescription items, such as antihistamines, for example chlorpheniramine, supplements, nootropics and herbal products) to help them sleep.

**TABLE 2:** Clinical profile of participants (*N* = 235).

Variable	*n*	%
**Insomnia**		
No (SCI > 16)†	137	58.3
Yes (SCI ≤ 16)†	98	41.7
**Family history of insomnia**		
No	168	71.8
Yes	66	28.2
**Psychiatric history**		
No	218	92.8
Yes	17	7.2
**Medical history**		
No	165	70.2
Yes	70	29.8
**Substance use**		
**Alcohol**		
No	206	88.0
Yes	28	12.0
Not reported	1	0.4
**Cannabis**		
No	221	99.1
Yes	2	0.9
Not reported	12	5.1
**Cups of caffeine per day**		
0	49	20.9
1–2	140	59.8
> 2	45	19.2
Not reported	1	0.4
**Nicotine**		
No	212	91.4
Yes	20	8.6
Not reported	3	1.3
**Used prescribed sedatives**		
No	187	79.6
Yes	48	20.4
**Used over-the-counter medications**		
No	150	63.8
Yes	85	36.2

† , SCI: Sleep Condition Indicator score

### Sleep hygiene

The results of the SHI showed that participants scored between 0 and 48 (µ = 18.37; s.d.: 8.46). Individuals suffering from insomnia had sub-optimal sleep hygiene (µ = 22.3; s.d.: 7.5) in comparison to those without insomnia (SHI µ = 15.55; s.d.: 8.0) (Table 3). Sleep Hygiene Index items that were significantly associated with insomnia were irregular sleeping and waking times; using alcohol or caffeine 4 h before bed; engaging in stimulating activities before bed; going to bed feeling stressed, angry, upset or nervous; using bed for activities other than sleep and/or sex and thinking, planning or worrying while in bed (Table 3). Environmental factors such as room and bed comfort were not associated with insomnia.

**TABLE 3:** Frequency of Sleep Hygiene Index items in participants with and without insomnia.

Sleep Hygiene Index items	Frequency	No insomnia†		Insomnia†		*P*
		*n*	%	*n*	%	
Daytime naps ≥ 2 h	Never/rarely/sometimes	109	62.6	65	37.4	0.020*
	Always/frequently	28	45.9	33	54.1	
Going to bed at different times	Never/rarely/sometimes	85	64.9	46	35.1	0.020*
	Always/frequently	52	50.0	52	50.0	
Getting out of bed at different times	Never/rarely/sometimes	98	63.6	56	36.4	0.002*
	Always/frequently	39	48.1	42	51.9	
Exercising before bed	Never/rarely/sometimes	128	56.9	97	43.1	0.040*
	Always/frequently	9	90.0	1	10.0	
Staying in bed longer than should	Never/rarely/sometimes	112	62.9	66	37.1	0.010*
	Always/frequently	25	43.9	32	56.1	
Using alcohol, tobacco or caffeine 4 h before bedtime	Never/rarely/sometimes	127	62.0	78	38.0	0.003*
	Always/frequently	10	33.3	20	66.7	
Engaging in stimulating activities before bedtime (e.g. video games, Internet)	Never/rarely/sometimes	103	68.2	48	31.8	< 0.001*
	Always/frequently	34	40.5	50	59.5	
Going to bed feeling stressed, angry, upset or nervous	Never/rarely/sometimes	124	63.9	70	36.1	< 0.001*
	Always/frequently	13	39.4	20	60.6	
Using bed for activities other than sleeping or sex	Never/rarely/sometimes	116	63.7	66	36.3	0.002*
	Always/frequently	21	39.6	32	60.4	
Uncomfortable bed (e.g. poor mattress or pillow)	Never/rarely/sometimes	123	59.1	85	40.9	0.470
	Always/frequently	14	51.9	13	48.1	
Uncomfortable room (e.g. too bright, stuffy, hot, cold or noisy)	Never/rarely/sometimes	121	59.3	83	40.7	0.420
	Always/frequently	16	51.6	15	48.4	
Doing important work before bedtime (e.g. paying bills, studying)	Never/rarely/sometimes	117	60.9	75	39.1	0.080
	Always/frequently	20	46.5	23	53.5	
Thinking, planning or worrying when in bed	Never/rarely/sometimes	98	73.7	35	26.3	< 0.001*
	Always/frequently	39	38.3	63	61.8	-
SHI score mean (s.d.)	-	15.55	(8.0)	22.3	(7.5)	-

SHI, Single-item measure of burnout; s.d., standard deviation.

* , Statistically significant (*p* < 0.05).

† , No insomnia = Sleep Condition Indicator score (SCI) ≤ 16, Insomnia = SCI > 16.

### Association between insomnia, sleep hygiene and socio-demographic, clinical and occupational factors

Univariate logistic regression demonstrated that the presence of insomnia was significantly associated with a positive family history of the condition (*p* = 0.001), a higher educational level (Masters/Speciality) (*p* = 0.03), the presence of medical and psychiatric disorders (*p* = 0.002), the regular use of alcohol and nicotine (*p* = 0.004 and 0.027, respectively), poor sleep hygiene (*p* < 0.001) and all categories of work-related burnout (*p* < 0.01) (Table 4). After controlling for covariates, only psychiatric history (aOR: 5.52; CI:1.06–28.66), all SIMB categories, inclusive of stress and burnout and sleep hygiene (aOR: 1.07; CI: 1.02–1.13) were significantly associated with insomnia, while 1–2 cups of coffee appeared to be protective (*p* = 0.02, aOR:0.36; CI: 0.15–0.38).

**TABLE 4:** Logistic regression: association between insomnia, socio-demographic, clinical, occupational variables and sleep hygiene.

Variable	SCI category					Odds of insomnia				Adjusted odds of insomnia			
	No insomnia†		Insomnia‡		Total	*p*	OR	95% CI		*p*	aOR	95% CI	
	*n*	%	*n*	%				Lower limit	Upper limit			Lower limit	Upper limit
**Gender**													
Female	103	56.6	79	43.4	182	-	1.00	-	-	-	-	-	-
Male	34	64.2	19	35.8	53	0.33	0.73	0.39	1.37	-	-	-	-
**Age (years)**													
22–30	13	61.9	8	38.1	21	-	1.00	-	-	-	-	-	-
31–40	48	61.5	30	38.5	78	0.98	1.02	0.38	2.74	-	-	-	-
41–50	51	56.7	39	43.3	90	0.66	1.24	0.47	3.29	-	-	-	-
51–60	25	54.3	21	45.7	46	0.56	1.37	0.48	3.92	-	-	-	-
**Marital status**													
Single or divorced	56	56.0	44	44.0	100	-	1.00	-	-	-	-	-	-
Married or cohabiting	72	60.0	48	40.0	120	0.77	0.85	0.28	2.56	-	-	-	-
Widowed	9	60.0	6	40.0	15	0.55	0.85	0.50	1.45	-	-	-	-
**Education level**													
Nursing certificate	32	69.6	14	30.4	46	-	1.00	-	-	-	-	-	-
Diploma or bridging	78	57.8	57	2.2	135	0.16	0.67	0.82	3.41	0.71	0.75	0.17	3.35
Bachelors	15	57.7	11	42.3	26	0.31	0.68	0.62	4.56	0.57	0.57	0.08	3.96
Masters or speciality	12	42.9	16	57.1	28	0.03	3.05	1.15	8.10	0.71	1.43	0.22	9.21
**Occupational category**													
Enrolled nurse assistant	33	67.3	16	32.7	49	-	1.00	-	-	-	-	-	-
Staff nurse	17	68.0	8	32.0	25	0.96	0.97	0.35	2.72	0.32	0.46	0.10	2.11
Professional nurse	76	53.9	65	46.1	141	0.10	1.76	0.89	3.49	0.73	1.29	0.31	5.34
Manager	11	55.0	9	45.0	20	0.34	1.69	0.58	4.89	0.76	0.72	0.09	5.76
**Work environment**													
Inpatient	105	60.7	68	39.3	173	-	1.00	1.00	-	-	-	-	-
Outpatient	24	58.5	17	41.5	41	0.08	1.09	0.55	2.19	0.24	2.87	0.50	16.50
Office	5	35.7	9	64.3	14	0.80	2.78	0.89	8.65	0.57	0.75	0.27	2.05
**Employment duration**													
≤ 24 months	16	50.0	16	50.0	32	-	-	-	-	-	-	-	-
> 24 months	117	59.4	80	40.6	197	0.32	0.68	0.32	1.45	-	-	-	-
**Night shift**													
No	36	57.1	27	42.9	63	-	-	-	-	-	-	-	-
Yes	96	57.8	70	42.2	166	0.90	0.97	0.54	1.75	-	-	-	-
**Family history**													
No	110	65.5	58	34.5	168	-	1.00	-	-	-	-	-	-
Yes	27	40.9	39	59.1	66	0.001	2.74	1.53	4.92	0.44*	1.38	0.61	3.11
**Psychiatric history**													
No	134	61.5	84	38.5	218	-	1.00	-	-	-	-	-	-
Yes	3	17.6	14	82.4	17	0.002	7.44	2.08	26.7	0.04	5.52	1.06	28.66
**Medical history**													
No	107	64.8	58	35.2	165	-	-	-	-	-	-	-	-
Yes	30	42.9	40	57.1	70	0.002	2.46	1.39	4.35	0.35*	1.46	0.66	3.23
**Alcohol use**													
No	127	61.7	79	38.3	206	-	-	-	-	-	-	-	-
Yes	9	32.1	19	67.9	28	0.004	3.39	1.46	7.87	0.27*	1.95	0.59	6.41
**Cups of caffeine per day**													
0	22	44.9	27	55.1	49	-	1.00	-	-	-	-	-	-
1–2	96	68.6	44	31.4	140	0.004	0.37	0.19	0.73	0.02	0.36	0.15	0.83
> 2	18	40.0	27	60.0	45	0.632	1.22	0.54	2.78	0.43	0.64	0.21	1.95
**Nicotine**													
No	130	61.3	82	38.7	212	-	1.00	-	-	-	-	-	-
Yes	7	35.0	13	65.0	20	0.027	2.94	1.13	7.69	0.68*	1.36	0.31	6.00
**Burnout category**													
5 –No burnout	55	90.2	6	9.8	61	-	1.00	-	-	-	-	-	-
4	39	63.9	22	36.1	61	0.001	5.17	1.92	13.9	0.02	3.86	1.19	12.48
3	22	37.9	36	62.1	58	< 0.001	15.00	5.54	40.6	< 0.01	11.1	3.48	35.43
2	16	43.2	21	56.8	37	< 0.001	12.03	4.15	34.9	0.00	7.19	2.05	25.23
1–Total burnout	5	27.8	13	72.2	18	< 0.001	23.83	6.29	90.3	0.00	10.3	2.25	46.89
Sleep Hygiene Index	137	-	98	-	-	< 0.001	1.12	1.07	1.16	0.01	1.07	1.02	1.13

SCI, Sleep Condition Indicator; s.d., standard deviation; OR, odds ratio; Cl, confidence level; aOR, adjusted odds ratio.

† , No insomnia: Mean SCI score = 15.55; s.d. = 8.0;

‡ , Insomnia: Mean SCI score = 22.3; s.d. = 7.5.

* , No longer statistically significant in the regression model.

## Discussion

The study aimed to determine the burden and factors associated with insomnia amongst nursing staff in public health facilities in KZN. The findings that 41.7% of nurses met the criteria for a DSM-5 diagnosis of insomnia disorder are concerning but in keeping with a recent systematic review that showed insomnia rates between 12.8% and 76.4% in health care workers.^28^ The results are similar to a Kenyan study, which showed a prevalence of 41% in health care workers.^9^ An international systematic review showed a prevalence of 43% in nurses during the COVID 19 pandemic,^29^ while in South Africa data are lacking. The consistent high prevalence rates of insomnia in nurses call for targeted interventions to improve sleep hygiene practises, well-being and better working conditions.

Our findings may be attributed to the predominance of females in our sample, as only 22.6% of our sample were male, matching the gender profile of the nurses employed by the South African Department of Health^30^ There was no significant difference between the males and females in our sample. In multiple studies, females with insomnia outnumbered males,^31^ which may be because of women’s higher prevalence of depression and anxiety disorders, which are strong insomnia risk factors.^31^ We explored the relationship between insomnia and multiple risk factors and found no significant association between covariates such as age and gender and insomnia, which is inconsistent with previous research.^3^ Future research should therefore consider targeted sampling and particular focus on male staff, who may be at increased risk in the SA context.

Insomnia in our study was associated with a family history, which is in keeping with other studies that show it to have a genetic basis.^32^ Additionally, most mental disorders have a genetic component,^33^ and while we did not specifically investigate for a family history of mental illness, we found that a diagnosis of mental illness was significantly associated with insomnia in our sample.

Insomnia was correlated with a higher degree of education, which contradicted the findings of an earlier study where insomnia was reported to be lower in people with a higher level of education.^34^ This may be attributed to the increased level of responsibility that typically accompanies higher positions in a short-staffed nursing hierarchy. Chronic medical illnesses such as metabolic syndrome, and psychiatric disorders, e.g. mood disorders, were associated with insomnia in our sample. This aligns with the prevailing research, which indicates that insomnia frequently co-occurs with medical and psychological disorders, necessitating proper diagnosis and treatment.^35^

Our study also revealed an association between nicotine use and the occurrence of insomnia, and while consuming more than two cups of caffeinated drinks was not associated with poorer sleep patterns in our sample, 1–2 cups were protective. Research has indicated that nicotine has the potential to induce insomnia, and in turn, insomnia can lead to the use of nicotine and other substances as a technique for self-medication.^36^ While both substances are psychostimulants, caffeine, an adenosine antagonist, temporarily lowers sleep pressure via affecting the sleep–wake rhythm. However, some studies have shown that low quantities of caffeine, for example as found in green tea, may have a sleep-promoting effect by reducing stress and upregulating adenosine receptors.^37,38^ However, caffeine use before bedtime and the high quantities of caffeine per drink have been shown to impair sleep in multiple studies.^37^ Our findings could be because of some participants’ tolerance to caffeine and the time over which the coffee is consumed. It is also possible that participants with pre-existing insomnia had reduced their caffeine intake, therefore reflecting a lower consumption pattern. Longitudinal studies would be more appropriate to unpack the association between both nicotine and caffeine.

An unexpected finding in our sample was that shift work was not associated with insomnia, which could be because of the nurses in the public sector working shifts for many months, thus acclimatising to a new circadian rhythm. Systemic reviews have shown that changing shifts disrupts the circadian rhythm and results in insomnia.^28^ Most of the nurses in this study experienced different levels of work-related burnout, and our finding that this is significantly associated with insomnia, when controlling for other factors, mirrors other studies,^39,40^ and underscores the importance of promoting health care worker wellness and addressing work-related stressors.

Our findings that nurses suffering from insomnia had inadequate sleep hygiene practices is consistent with recent research findings,^41^ with poor sleep hygiene possibly being caused by shift work and poor sleep education.^42,43^ Almost all sleep hygiene practices, besides environmental comfort were associated with better sleep in our study, which highlights the importance of promoting positive wellness practices, not just on the patients they serve but also among health care workers themselves as a simple and cost-effective strategy.

A few limitations may have affected our findings, the cross-sectional design being unable to establish causality, the questionnaires being self-rated and depended on the participants’ recall and the findings therefore possibly being over- or under-reported. Furthermore, alcohol, cannabis and nicotine use were also self-reported as binary responses and not quantified. The questionnaires used were not validated in South Africa, according to the author’s knowledge, although they have been used internationally. The study’s strength lies in its modest sample size and its focus on insomnia disorder as defined in the DSM-5, which necessitates functional impairment, while simultaneously emphasising the impact of burnout on sleep. While similar data may exist for other countries, replicating this study in South Africa is important for addressing a notable gap in local data, given the region’s unique socio-economic and cultural dynamics that may influence the interplay between burnout and insomnia. As the study was only conducted in KZN, the findings may not reflect the broader community of nurses in other provinces. Future studies would be benefit with using non-self-report measures of insomnia, such as smart watches for more objective data on sleep patterns and to focus on interventions.

## Conclusion

The study expands on the emerging literature on health care worker wellness in SA by focusing on insomnia and possible associations, because of it being an important component of physical and mental wellness. The high rate of insomnia raises awareness about its effects on the quality of nursing and the personal burden related to a lack of sleep among public sector nurses who often work in under-staffed and under-resourced circumstances. Such studies can serve as a reference for future health and wellness promotion aimed at health care professionals. Assisting nurses to have good quality sleep is likely to result in their having more job satisfaction and being able to provide optimal patient care.
